# DendroScan: an open source tool to conduct comparative statistical tests and dendrogrammatic analyses on particle morphometry

**DOI:** 10.1038/s41598-020-78698-0

**Published:** 2020-12-10

**Authors:** T. Dürig, L. S. Schmidt, J. D. L. White, M. H. Bowman

**Affiliations:** 1 Geology Department, University of Otago, Dunedin, New Zealand; 2 Department of Geosciences, University of Oslo, Oslo, Norway; 3 Present Address: Institute of Earth Sciences, University of Iceland, Reykjavík, Iceland

**Keywords:** Solid Earth sciences, Geology, Volcanology, Techniques and instrumentation, Software, Statistics

## Abstract

Quantitative shape analysis of juvenile pyroclasts is applied in volcanology to reconstruct the dynamics and styles of eruptions, and to explore the details of tephra transport, dispersal, and emplacement. Morphometric analyses often include comparison of multiple data sets with a set of dimensionless shape parameters. Here we present “DendroScan”, an open source Matlab program that provides the user with all the multivariate statistical methods needed to produce such morphometric comparisons. Serving as a statistical “toolbox”, DendroScan conducts Levene-, t-, and equivalence tests, presenting the results in ad hoc interpretable graphs. Furthermore, it is designed to conduct dendrogrammatic analyses of particle morphometry, a recently developed approach for the inter-comparison of multiple morphometric data sets. DendroScan produces tree diagrams, in which the analysed samples are sorted according to their morphometric dissimilarity, allowing the user to identify, e.g., samples that are statistically equivalent. To demonstrate DendroScan’s potential, ten experimental samples are compared with volcanic ash samples generated by the Havre 2012 deep-sea eruption in the Kermadec arc (New Zealand). We show how, using DendroScan-based results, information on the eruptive mechanism can be inferred, and how the cooling history of the experimental melt is reflected in the dissimilarity of thermally granulated fragments.

## Introduction

Quantitative analysis of juvenile pyroclast shapes, known as “morphometric analysis”, is a technique commonly used in volcanology to infer the style of unwitnessed eruptions^1–7^ and specific characteristics of particle-forming processes^8–11^. Particle shape analysis is used to study the modes of tephra transport, dispersal and emplacement^12–18^ and to provide insights in eruption dynamics during magma fragmentation. e.g., ^19–24^.

Starting with a qualitative characterization of ash particles e.g., ^7,25–27^, particle morphometry has evolved over the last quarter century, and various interpreter-independent systems have been suggested to quantitatively describe either the projected shapes or the cross-sections of ash particles in two dimensions. The five morphometric systems most commonly used in volcanology^2,28–31^ have been implemented in recently published open-source software PARTISAN (PARTIcle Shape ANalyzer), which computes 23 dimensionless shape parameters of binarized 2D objects^32^.

Morphometric comparison of one sample to another is a relatively easy statistical task, which can be solved, for example, by the application of t-tests^3,8,33^, but significantly more complications arise when multiple samples have to be compared with one another^34^. Mathematically, such multiple comparisons would require use of numerous stochastically drawn subsets of samples, which, because this would require large sample sizes, is often impractical. In order to counter this difficulty a strategy has been proposed, labeled “dendrogrammatic analysis of particle morphometry” (DAPM), which involves the application of a number of different statistical tests^34^. In this article, we suggest a further refinement of the DAPM and present an open source Matlab program, baptized “DendroScan”, which assists the user with performing comparative statistical analyses among multiple morphometric data sets. The latter term describes any *n* × *m* matrix of *n* samples, whose shapes are quantified by *m* parameters^34^. DendroScan runs on any platform supported by Matlab and provides the user with all the statistical tools required for comparative analysis of morphometric datasets, including one-way analysis of variances (ANOVA), t-tests, equivalence tests (e-tests) and DAPM. In DendroScan, each of these tests can be executed either manually or automatically, following the suggested DAPM protocol. The results are visualised in descriptive dendrograms and bar plots.

Previous studies on volcanic ash of the submarine Havre 2012 eruption have revealed that it was produced by a variety of different ash generation mechanisms^34–36^, including a novel thermohydraulic fragmentation process termed IFCI (induced fuel coolant-interaction)^36^. Below we compare two ash samples from this remote deep-sea volcano with ten samples generated in various melt fragmentation experiments in order to investigate the effects of changes in the experimental conditions on particle morphometry and to demonstrate the capabilities of DendroScan.

## Statistical analysis methods implemented in DendroScan

DendroScan reads “.csv” data files as provided by the particle-shape-analysis software PARTISAN^32^. The ".csv" file comprises a list of scanned grains and their corresponding shape parameters (listed in Table 1). Although it is recommended that the companion PARTISAN software is used to prepare the “.csv” file, it is not strictly necessary. As long as the column order is respected the user may create a “.csv” file with their own morphological calculations, using the provided example “.csv” files (see Supplementary Data) as a template.

**Table 1:** Notation of shape parameters and respective morphometric systems analysed by DendroScan. Shape parameters which are mathematically equivalent and therefore would provide redundant information, are omitted by default for DendroScan analyses, but could nevertheless be activated by the user. Definitions for each shape parameter are provided in the second column, with *p* particle perimeter, *A* projected particle area, *w* short side of the minimum area bounding rectangle, *b* long side of the minimum area bounding rectangle, *c* perimeter of the circle with area *A*, *a* maximum intercept, *m* mean intercept perpendicular to *a*, *L*_*b*_ maximum length of all possible lines from one point of the perimeter to another point on the perimeter projected on the major axis of the particle, *W*_*b*_ maximum of all possible lines from one point of the perimeter to another point on the perimeter, projected on the minor particle axis, *p*_*cp*_ perimeter of smallest convex polygon around particle, *A*_*cp*_ area of smallest convex polygon around particle, *e*_*ce*_ perimeter the smallest area ellipse that encloses, but does not intersect the particle, L_maj_ major axis of best fit ellipse, *L*_*min*_ minor axis of best fit ellipse, *d*_*BC*_ diameter of circle that encloses, but not intersects particle, *l*_*F*_ minimum Feret length, *w*_*F*_ Feret length perpendicular to *l*_*F*_ and *d*_*F*_ maximum Feret distance. For further details see Table 1 in Dürig et al. ^32^.

Notation	Definition	Shape parameter	Used by default	Remark	Morphometric system by
*Circ_DL*	$p/c$	Circularity	Yes		Dellino and La Volpe^2^
*Rec_DL*	$p/\left( {2b + 2w} \right)$	Rectangularity	Yes		
*Com_DL*	$A/\left( {b \cdot w} \right)$	Compactness	Yes		
*Elo_DL*	$a/m$	Elongation	Yes		
*Circ_CI*	*4* $\pi A/p^{2}$	Circularity	Yes		Cioni et al.^29^
*AR_CI*	$L_{maj} /L_{min}$	Aspect ratio	Yes		
*Con_CI*	$e_{ce} /p$	Convexity	Yes		
*Sol_CI*	$A/A_{CP}$	Solidity	Yes		
*Circ_LL*	$c/p$	Circularity	Yes		Leibrandt and Le Pennec^28^
*Elo_LL*	$1 - W_{b} /L_{b}$	Elongation	Yes		
*AR_LL*	$W_{b} /L_{b}$	Aspect ratio	Yes		
*Con_LL*	$p_{CP} /p$	Convexity	Yes		
*Sol_LL*	$A/A_{CP}$	Solidity	No	Identical to *Sol_CI*	
*FF*	*4* $\pi A/p^{2}$	Form factor	No	Identical to *Circ_CI*	Liu et al. ^31^
*AR_LI*	$L_{min} /L_{maj}$	Aspect ratio	Yes		
*Con_LI*	$p_{CP} /p$	Convexity	No	Identical to *Con_LL*	
*Sol_LI*	$A/A_{CP}$	Solidity	No	Identical to *Sol_CI*	
*Circ_SC*	$4A/\pi d_{BC}^{2}$	Circularity	Yes		Schmith et al. ^30^
*Rec_SC*	$A/\left( {b \cdot w} \right)$	Rectangularity	No	Identical to *Com_DL*	
*FF_1*	*4* $\pi A/p^{2}$	Form factor	No	Identical to *Circ_CI*	
*AR_F*	$l_{F} /w_{F}$	Feret aspect ratio	Yes		
*AR_SC*	$w_{F} /l_{F}$	Reciprocal aspect ratio	Yes		
*Reg*	$16A^{3} /\left( {b \cdot w \cdot p^{2 \cdot } d_{BC}^{2} } \right)$	Regularity	Yes		

DendroScan permits analyses based on any of the 23 shape parameters, but since there is repetition of some of them across different systems, only 17 are suggested for comparative analyses^32^. Testing two samples (files) would therefore involve the pairwise comparison of the distributions of 17 shape parameters.

### T-tests

Based on the Student’s t-distribution^37,38^, t-tests have been previously applied in comparative analyses of particle shape^8,11,33^. A t-test compares two data sets and computes the error likelihood (“*p* value”) of rejecting the null hypothesis, which states that the tested data sets are from the same population. If the error likelihood *p* is below the level of significance α, the null hypothesis can be rejected: the data sets are then verified to be “significantly different” in the tested hypothesis^11,39^.

Before a t-test is applied, DendroScan checks whether the variances of the data sets are homogeneous by running a Levene test. In cases where the variances of the compared data sets are verified to be homogeneous, the results of a “pooled variance t-test”^39^ are used. If this precondition is not met, however, a “separated variance t-test”^40^ is conducted.

This provides a robust method to test two sets of randomly selected samples, but the reliability of a t-test is reduced when the same data sets are repeatedly used^41^. As a consequence, the likelihood of a type I error (test indicates a significant difference where there is none) increases.

Post-hoc adjustments could counter this effect, e.g. the Bonferroni correction^42^, but at the price of reducing statistical power^41,43^ and increasing the likelihood of type II errors, where genuine differences are no longer detected by the test.

### One-way analysis of variances (ANOVA)

In contrast to t-tests, ANOVA is based on the Fisher-Snedecor probability distribution, also known as “F-distribution”^44^, and is applied where there are more than two data sets at once to be analysed^39^. Similar to t-tests, the effect of increased type I error has to be adjusted for, by the application of post-hoc corrections, whenever data sets are repeatedly tested.

Again, Levene-tests serve to check the homogeneity of the data sets. Depending on test outcomes, DendroScan uses ANOVA to compute the *p* values and subsequently adjust them using one of two post-hoc corrections:

1. Tukey’s range test (also known as Tukey honestly significant difference HSD) is applied as post-hoc correction for assumed homogeneous variances^45^.
2. Games-Howell post-hoc adjustment^46^ is used for samples with heterogeneous variances.

### Refined equivalence tests (“e-tests”)

While ANOVA and t-tests are designed to prove significant differences, they cannot be used to verify if two data sets are “statistically equivalent”^11,47^. This is the purpose of equivalence tests (“e-tests”), which were introduced by Dürig et al.^11^ for morphometric analyses. This method tests whether the confidence interval *Δ* (with level of significance being *α*) of a shape parameter from one sample is within a given acceptable range *D*_*max*_, denoted “equivalence margin”^48,49^, which specifies a lower and an upper boundary *Δ*_*L*_ and *Δ*_*U*_. To verify data congruence, two one-sided t-tests are conducted, one of either side of the equivalence margin, testing the composed null hypotheses *H*_*01*_*: Δ* < *Δ*_*L*_ and *H*_*02*_*: Δ* > *Δ*_*U*_. T-test results leading to the rejection of both hypotheses imply that *Δ*_*L*_ < *Δ* < *Δ*_*U*_ and serve as proof for statistical equivalence^48,49^.

In previous studies, equivalence tests were exclusively based on the pooled Student’s t-function, and therefore provided reliable results only for data sets having homogeneous variances^11,34,36^. For DendroScan, however, e-tests were refined by implementing also one-sided “separate variance” t-tests, based on Welch’s adjusted t-function for data sets with heterogenous variances^40^. As for t-tests and ANOVA post-hoc corrections, the results of Levene-tests are used to decide which of the two t-functions is applicable.

The equivalence margin is shape-parameter and case-specific^11^ and usually determined by applying calibration tests using a number of samples which are drawn from the same population and therefore are known to be statistically equivalent^34,36^.

In these calibration tests, DendroScan reiteratively computes e-tests for each shape parameter, starting with a *D*_*max*_ value of 0.01 and increasing it stepwise by 0.01, until a statistical equivalence is indicated.

### Dendrogrammatic analysis of particle morphometry (DAPM) and statistical power index (SPI)

Following a recent study^34^, DendroScan uses the ANOVA-based *p* values to construct a matrix *X* with the elements:

1 $$X_{ij} = \mathop \sum \limits_{k = 1}^{m} Y_{ijk}$$

with *p*_*ijk*_ being the *p* value of data set *i* tested with the one from data set *j* in the *k*-th of *m* shape parameters, and with *Y*_*ijk*_ being defined by:

2 $$Y_{ijk} = \left\{ {\begin{array}{*{20}l} {log\left( {1 + \frac{1}{{p_{ijk} }}} \right)} \hfill & {if\;p_{ijk} < 0.05 } \hfill \\ 0 \hfill & {if\;p_{ijk} \ge 0.05} \hfill \\ \end{array} } \right.$$

Using *X* as a distance matrix DendroScan then draws a dendrogram which visualises the relative morphometric differences between the tested data sets by grouping them in clusters.

The number of data sets *N* analysed correlates negatively to the statistical power of ANOVA with post-hoc corrections^34^. The larger *N*, the lower the likelihood that all differences between their shape parameters are reflected in the output. When analysing larger numbers of data sets (N > 7), it is therefore recommended to repeat the above described computation of *X* with reduced *N*. With DAPM, the suggested strategy is to level-wise reiterate the dendrogram analysis for data subsets within identified clusters, until no further change is observed^34^.

DendroScan provides a color-coded statistical power index (SPI) bar, which indicates if a repetition with lower *N* might be necessary. The SPI values range between 0 and 100, and are computed by:

3 $$SPI = 100 \cdot f\left( N \right)/f\left( 2 \right)$$

where *f* is the probability density function:

4 $$f\left( N \right) = \frac{{exp\left( { - \left( {N - {\upmu }} \right)/s} \right)}}{{s \cdot \left( {1 + exp\left( { - \left( {N - {\upmu }} \right)/s} \right)} \right)^{2} }}$$

and *N* is the number of data sets, with the average *µ* and the standard deviation *s* being set to 2.4 and 4.5, respectively.

According to the DAPM protocol^34^, data sets which are grouped together with a dissimilarity of 0 in dendrograms of high SPI are subsequently analysed pairwise by two-tailed t-tests. In the final step, for samples which “fail” the t-tests (no significant differences found in any of the tested shape parameters), morphometric equivalence is verified by e-tests using the according threshold values *D*_*max*_.

With DendroScan, a DAPM can be conducted either automatically, or manually by following the above suggested steps. Below we will demonstrate both modes.

## Samples used for demonstration

### Natural ash particles

The volcanic ash samples studied were produced in the 2012 eruption of Havre, a silicic submarine volcano in the Kermadec Arc, at a depth of ~ 1000 m below sea level^35,50^. The samples were retrieved during an expedition in 2015 at six different locations^34^ and were sorted by four morphological classes (curvi-planar, angular, elongate tube and fluidal), as suggested by Murch et al.^35^. Five samples of curvi-planar grains (denoted CALcp_I, …, CALcp_V) and four samples of angular grains (denoted CALang_I, …, CALang_IV) contained 20 particles or more, and serve as “standards”: these samples are used to calibrate the equivalence margins *D*_*max*_ in the e-tests and are identical to the data sets used for the same purpose in previous studies on Havre^34,36^.

Subsequently, to demonstrate the ability of DendroScan to make comparisons, two data sets were randomly obtained from the six ash samples binned by morphological class, following the procedure described in Dürig et al.^34^:

- sample NATang: Havre ash with overall angular (jagged) morphology.
- sample NATcp: Havre ash with overall curvi-planar (blocky) morphology.

### Experimental particles

Experimental particles were produced under laboratory conditions by conducting fragmentation experiments using remelted Havre rock and pumice (see also list in Table 2).

**Table 2:** List of samples used for DAPM demonstration using DendroScan.

Notation	Notation in literature^34^	Sample type	Melt material	Details of experiments	Sampling location
B		Experimental	Pumice	“Indent run”: similar to a ‘dry’ run, but a few seconds before the run, an indentation was made in the centre of the melt plug by using a poker	Adjacent water bowl
D		Experimental	Pumice	“Indent run”	On ground
F	PifciU	Experimental	Pumice	IFCI run with U-tube	Water bowl
Cair		Experimental	Dome rock	Contraction run; cooled in air	Crucible
Cinter		Experimental	Dome rock	Contraction run; cooled in air, with an interim water-cooling period after 120 s (600 ml water)	Crucible
Cstart		Experimental	Dome rock	Contraction run; initially cooled with 240 ml of water, subsequently in air	Crucible
Cwater		Experimental	Dome rock	Contraction run; completely water cooled	Crucible
U	RifciU	Experimental	Dome rock	IFCI run with U-tube; first ejecta phase	Water bowl
V		Experimental	Dome rock	IFCI run with U-tube; late ejecta phase	On ground
R		Experimental	Dome rock	‘Ramp run’: gas pressure was gradually increased until plug was deformed and fragmented	Adjacent water bowl
NATang	NatIang	Natural	–	Angular Havre ash	At Havre volcano
NATcp	NatIIcp	Natural	–	Curvi-planar Havre ash	At Havre volcano

For each run, 250 g of raw Havre material was granulated and remelted in a 10 cm diameter cylindrical steel crucible, using either pumice or rhyolitic dome rock as starting material^34^. After being inductively heated to 1573 K the melt was kept at this temperature for 30 min to equilibrate, then cooled for 30 min, until the experimental temperature of 1423 K was reached. Although the experiments were conducted at ambient pressure, while the eruptive processes at Havre occurred at a pressure of ~ 10 MPa, matches between laboratory and natural particles, and clear distinction of particles from water-involved fragmentation from dry ones, give us confidence that the type of eruptive process can still be determined by morphometric analysis^36^.

Four main types of melt fragmentation experiments were conducted:

“dry indent run” (samples D and B): the setup used for this type of experiment was based on standardized stress-induced fragmentation tests, where highly pressurized gas is injected into the melt from below^51,52^. In contrast to the standard procedure used in earlier studies^34,36^, ten seconds prior to gas release the melt plug was indented in its center by using a 15 mm diameter poker, in order to locally weaken the melt. Then argon was injected at 8.5 MPa by opening a solenoid valve. Expansion of the released argon gas overloaded the cylindrical plug, which deformed and fractured in a brittle way. Only pumice was used as starting material for dry indent runs. Two types of samples were retrieved on dry indent runs:

- sample B: particles sampled in a bowl filled with 600 ml of deionized water, which was located adjacent to the crucible;
- sample D: particles retrieved on the (dry) lab floor.

“ramp run” (sample R): for this experiment an identical setup was used as for the dry indent run, except that the melt was not indented beforehand. The most important difference, however, was that the ramp run was conducted with open solenoid by gradually opening the gas bottle valve, thus gradually increasing the gas pressure, until fragmentation occurred.

“IFCI runs” (samples F, U and V): Experiments which yielded induced fuel coolant-interaction (IFCI) used a similar setup as the one for dry indent runs, with the addition of a hose leading to the top of the crucible^36,53^. Raw material for the melt was either pumice or dome rock. In contrast to the dry indent runs, the melt was not indented prior to the run. Two seconds before gas injection, a 240 ml water layer was added on top of the melt. When the expanding argon initiated stress-induced material failure, water entered the opening cracks and started downward-advancing IFCI that thermo-hydraulically “boosted” fragmentation^36^. Because water entered cracks from the top, the leading front of the ejected cloud of fragments contained more thermo-hydraulically produced fragments (termed “IFCI particles”) than the following ejecta^36^. Along with water and steam, small particles of the leading ejecta front were guided into a bowl of deionized water via a 10 cm-diameter U-shaped steel tube. When larger fragments of the following ejecta entered the tube (typically, ~ 30 ms after initiation of fragmentation), their impact momentum pushed it upward and removed it from the particle-ejection path. Fragments ejected at this stage were deposited across the whole experimental area after following free ballistic trajectories. Further details of the IFCI setup with U-tube are provided in Dürig et al.^36^. Three different samples are considered for this demonstration:

- sample F: particles from IFCI runs with remelted pumice, retrieved in the water bowl via U-tube, representing grains from the leading ejecta front;
- sample U: particles from IFCI runs with remelted dome rock, retrieved in the water bowl via U-tube, representing grains from the leading ejecta front;
- sample V: particles from IFCI runs with remelted dome rock, retrieved on the lab floor; according to the considerations above, these fragments are assumed to be from the late ejecta phase, after the U-tube separated.

“Crucible contraction runs”: In these experiments remelted dome rock was kept inside the crucible and the 'plug' cooled to room temperature, using air and/or water. The fracture-mechanical properties of silicate melts show complex changes at the solid-ductile boundary^54^. During cooling, fields of mechanical stress are built up in the melt, which affect the formation and thus the shapes of fragments^10^. The steel crucible contracts faster during cooling than does the solidifying melt, so the crucible exerts radial compressional pressure onto the plug, fragmenting it. These experiments mimic thermo-mechanical fragmentation, analogous to fragmentation processes of brittle crusts, e.g. during continued lava movement^55^. Crucible-contraction runs were conducted with four different cooling procedures, resulting in:

- sample Cair: from crucible contraction runs; exclusively cooled in free air
- sample Cstart: from crucible contraction runs; initially cooled with 240 ml of water until water was vaporized, subsequently cooled in air
- sample Cinter: from crucible contraction runs; cooled in air, interrupted by an intermediate water (600 ml) cooling period, which started 120 s after begin of cooling; when water was vaporized cooling continued in air
- sample Cwater: from crucible contraction runs; completely cooled with water.

**Figure 1:**
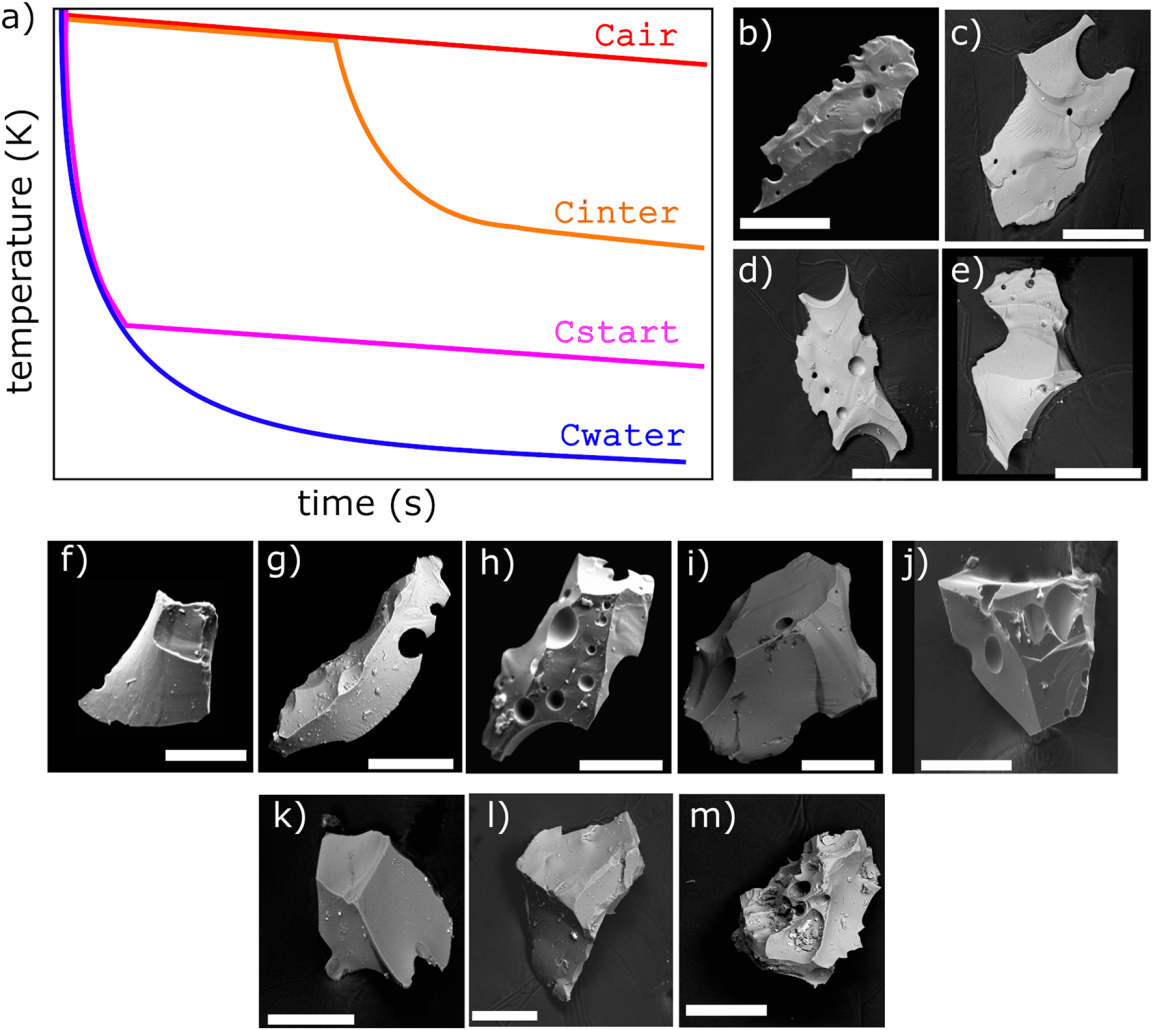
Experimental and natural particles used for demonstration. (**a**) Schematic cooling curves for the crucible-contraction run samples “Cair”, “Cinter”, “Cstart” and “Cwater”. SEM scans show typical particles from the analysed samples: (**b**) “Cair”, (**c**) “Cinter”, (**d**) “Cstart”, (**e**) “Cwater”, (**f**) “B”, (**g**) “D”, (**h**) “R”, (**i**) “F”, (**j**) “V”, (**k**) “U”, (**l**) “NATcp”, (**m**) “NATang”.

A schematic overview of the theoretical cooling curves in these runs is presented in Fig. 1a, and examples of particles of the four samples are shown in Fig. 1b–e.

Table 2 summarizes all samples used in this article for demonstration. Representative SEM scans are presented in Fig. 1b–m.

### Particle shape analysis

All samples were sieved in 1 phi steps. For morphometric analysis, particles of the 4 phi fraction (64–125 µm) were randomly selected and mounted on carbon-coated tape. A Zeiss Sigma VP FEG scanning electron microscope (SEM) provided backscatter electron scans with a resolution of 2048 × 1536 pixels.

After segmentation and binarization, particles were represented by black and white images (silhouettes). In the next step, these silhouettes were used as input data for the particle shape analyser software PARTISAN^32^. The resulting data files in csv format are labeled using the sample nomenclature above (e.g., NATang.csv”, “U.csv”) and provided along with this article (see Supplementary Data). Standard files for calibration are provided in two separate folders, labeled “curvi-planar”, and “angular”.

## Dendrogrammatic analysis using DendroScan

For demonstration, let our main aim here be an exploration into which of the experimental samples most closely match the natural ash samples “NATcp” and “NATang”.

DendroScan was tested for Matlab R2019b and does not require any additional toolboxes. The program is installed by unpacking the zip folder into a working directory. It is executed by running the script “main.m”. DendroScan reads .csv files produced by PARTISAN^32^.

### Step by step analysis (using manual test functions)

As a first step of DAPM, the ANOVA-based “level 1” dendrogram is generated, which considers all 12 samples to be analysed. To do this, the radio button for “dendrogram” in the “select test” field is selected. Then, a new field is displayed with a “load files” button that when clicked opens a file browser. After selecting the 12 sample files provided with this demonstration, a field allowing selection of shape parameters is unlocked. This demonstration uses the default setting, in which 17 shape parameters are selected (see Table 1). When one presses “OK” the X-matrix is computed and the dendrogram is plotted.

**Figure 2:**
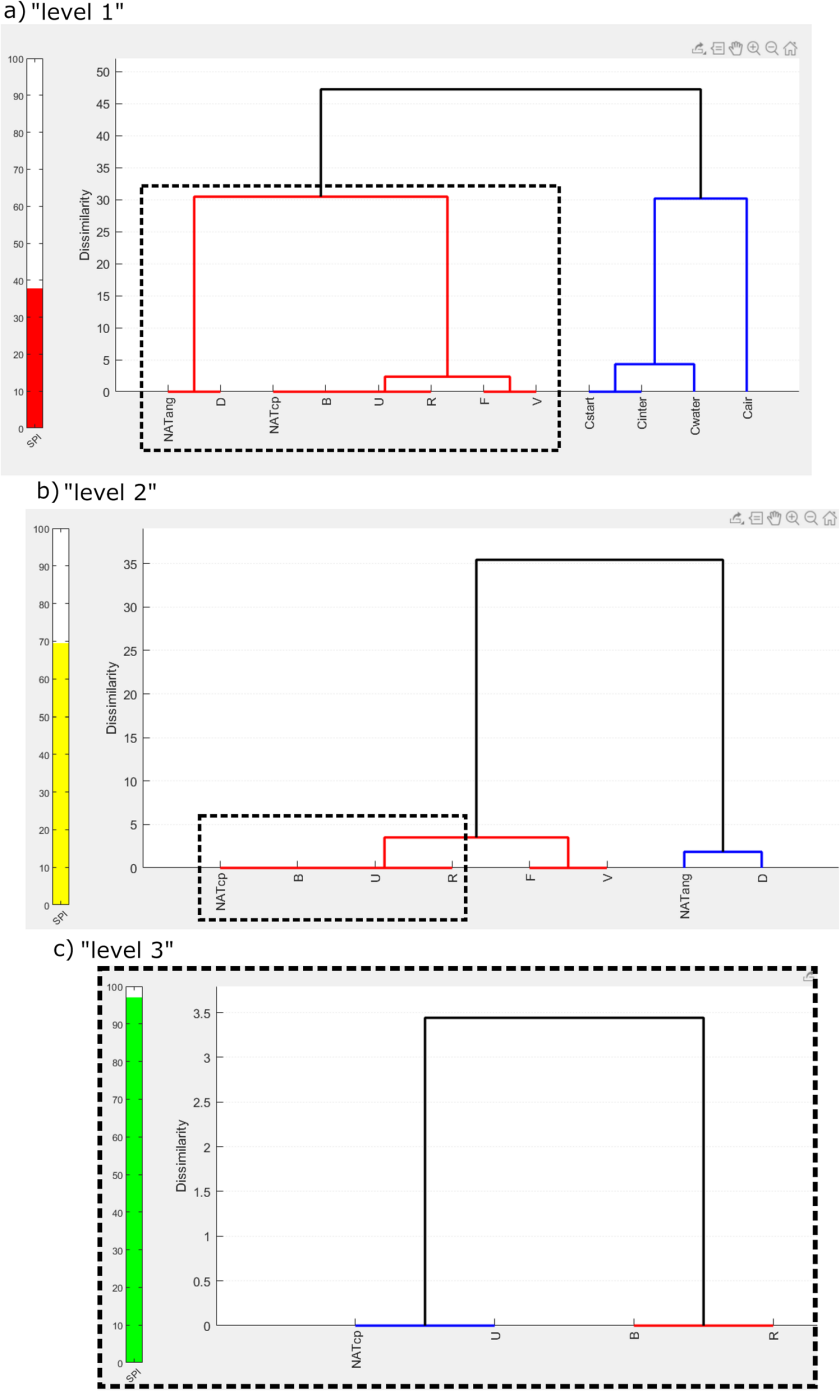
DendroScan screenshot, showing the dendrogram results for different data levels. (**a**) “Level 1” dendrogram in which all studied samples are considered. The red bar on the left indicates a low statistical power index (SPI). The 12 tested samples fall into two main clusters (marked by blue and red colour) and are separated by large dissimilarity values of more than 45. These two sample sets were individually used for further dendrogram computation. (**b**) To obtain the “level 2” dendrogram, all samples of the first main cluster (red group in a) were processed. The yellow SPI bar indicates moderate reliability. Dashed box encloses four samples suggested to be of identical morphology. (**c**) For these four samples a further-downsized (“level 3”) dendrogram was computed. The SPI is already quite high, but according to the DAPM protocol, further t- and e-tests have to be conducted before a statistical equivalence is ultimately verified.

The result is shown in Fig. 2a. Note that the SPI is only 38, and the red bar indicates a low reliability of the test-results upon which this dendrogram is based. This means that not too much trust should be put into results for samples with very low, or no, dissimilarities^34^. Nevertheless, two main clusters are separated by a dissimilarity value of over 45. One main cluster (marked in blue) comprises all crucible contraction runs (“C”) samples, while the rest are grouped in a different cluster. Each of these clusters is used for a subsequent individual (“level 2”) dendrogram computation.

The resulting “level 2” dendrogram of the latter main cluster is presented in Fig. 2b. Note that the samples D and NATang are no longer indicated to be of equivalent shape. (In fact, a t-test reveals significant differences in 10 of the 17 shape parameters between these two samples.) Already from this result, we can conclude that from all tested samples, particles produced in dry indent runs (“D” samples) were closest to the angular natural ash particles. Yet, their morphology is characterized by slight but detectable differences.

At this level, three samples are suggested as matching the morphology of curvi-planar Havre ash (“NATcp”): “B”, “U” and “R”. However, *N* was still too large to guarantee high reliability, indicated by a yellow SPI bar. In this case, best practice is to compute a “level 3″ dendrogram for only these four samples (Fig. 2c). The result groups “NATcp” together with “U”, and “B” together with “R”.

**Figure 3:**
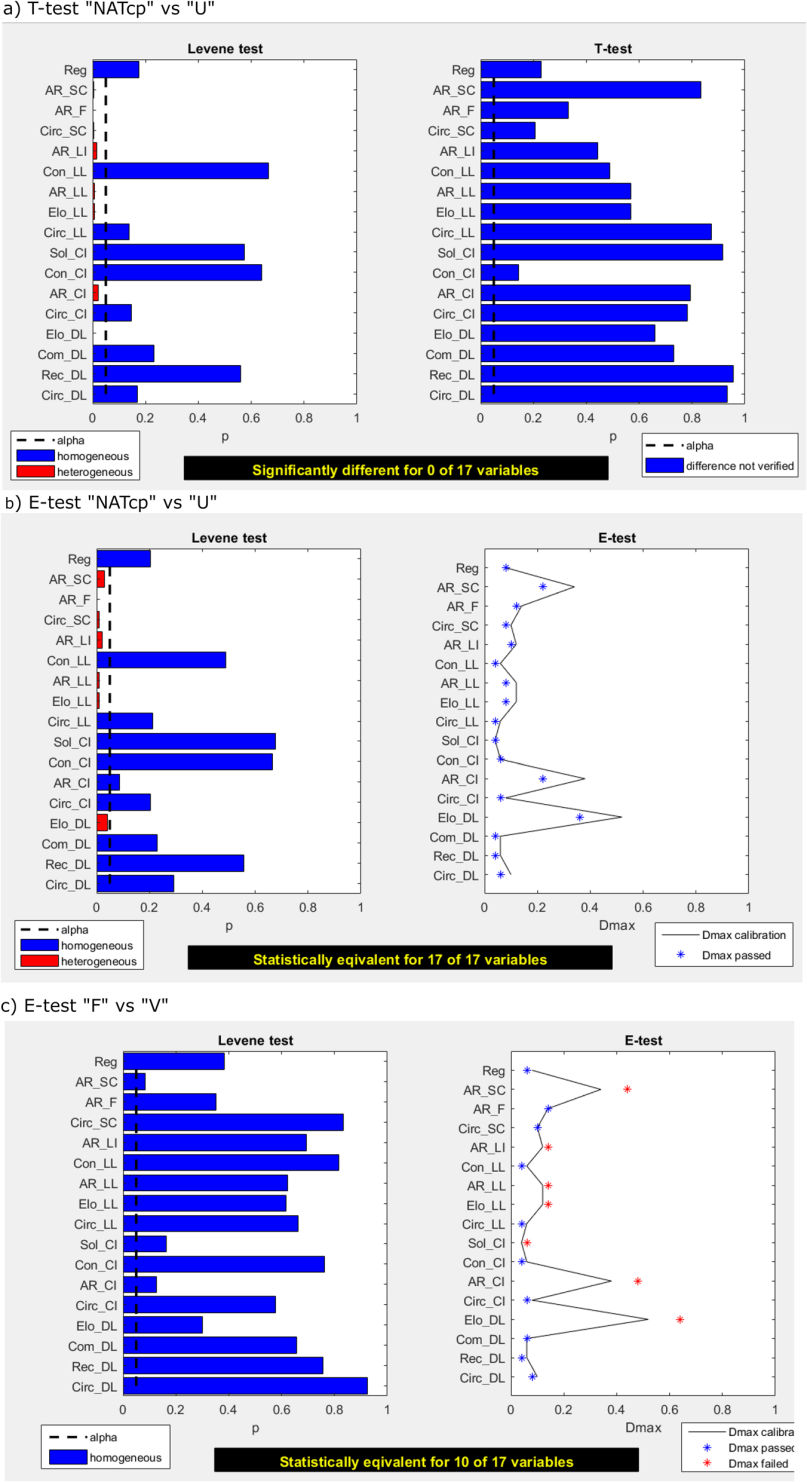
DendroScan screenshots displaying statistical test results. (**a**) Results of Levene tests (left) and t-tests (right) for natural ash sample “NATcp” tested versus experimental sample “U”. The left plot shows that Levene tests suggest homogeneous variances in 10 of the tested shape parameters. For the other 8 shape parameters, separate variance t-tests (“Welch tests”) were conducted. The right plot indicates no significant difference between “NATcp” and “U” for any of the 17 tested parameters. (**b**) DendroScan results for Levene-tests (left) and e-tests (right). The samples “NATcp” and “U” are verified to be statistically equivalent in all of the tested shape parameters. (**c**) In contrast, “F” and “V” show statistical equivalence for only 10 of the 17 shape parameters. Thus, these samples are still morphometrically distinguishable. The black line in the e-test plot represents the equivalence margin *D*_*max*_ and is dependent on the standards used for calibration.

Following the DAPM protocol, the next step foresees t-tests between the three sample pairs suggested by the dendrograms: “NATcp” versus “U”, “B” versus “R” and “F” versus “V”. Figure 3a provides an example, showing DendroScan’s t-test result window for the first sample pair. As for the other two comparisons, t-tests indicate no significant difference in any of the 17 tested shape parameters.

The suggested three sample pairs were subsequently tested with e-tests, using the provided standards for curvi-planar Havre ash (“CALcp_I”… “CALcp_V”), which is a calibration tailored for testing “NATcp”.

Figure 3b, c show two examples of how DendroScan displays the outcomes of e-tests: for each shape parameter, the computed values for *D*_*max*_ are plotted as markers, under which the e-test would be passed. The calibration-based equivalence margin is plotted as a black line. If the markers are left of the black line, it means that statistical equivalence can be assumed (also indicated by the blue color of each of the markers). In contrast, a red marker indicates that the deviation is too large to confirm the tested assumption.

According to e-tests, statistical equivalence is only verified for “NATcp” and “U”, but not for the other two data pairs.

**Figure 4:**
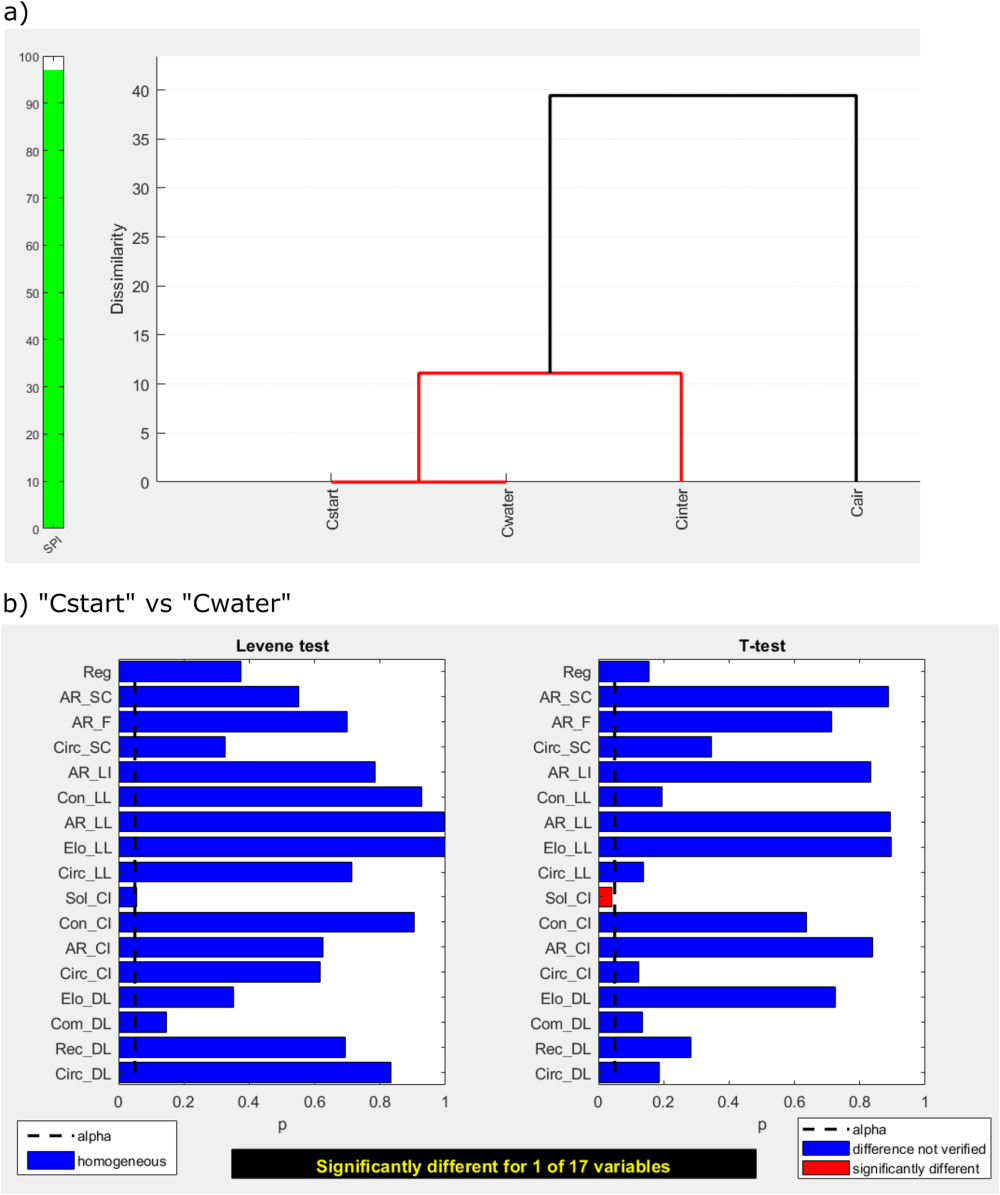
Analysis results for contraction-run samples (red group in Fig. 2a). The “level 2” dendrogram suggests a similarity between “Cstart” and “Cwater”. The t-tests reveal, however, a significant difference in one shape parameter (solidity), implying that “Cstart” and “Cwater” are morphometrically distinguishable.

Computation of the second “level 2” dendrogram, which considers only the group of contraction-run samples, yields the plot in Fig. 4a. Compared to Fig. 2a, the sorting of the samples was slightly rearranged in response to the larger SPI and the higher reliability of the underlying tests. While the “level 2” dendrogram suggests “Cwater” and “Cstart” to be similar, t-tests reveal a significant difference in solidity (Fig. 4b).

### Automatic DAPM

**Figure 5:**
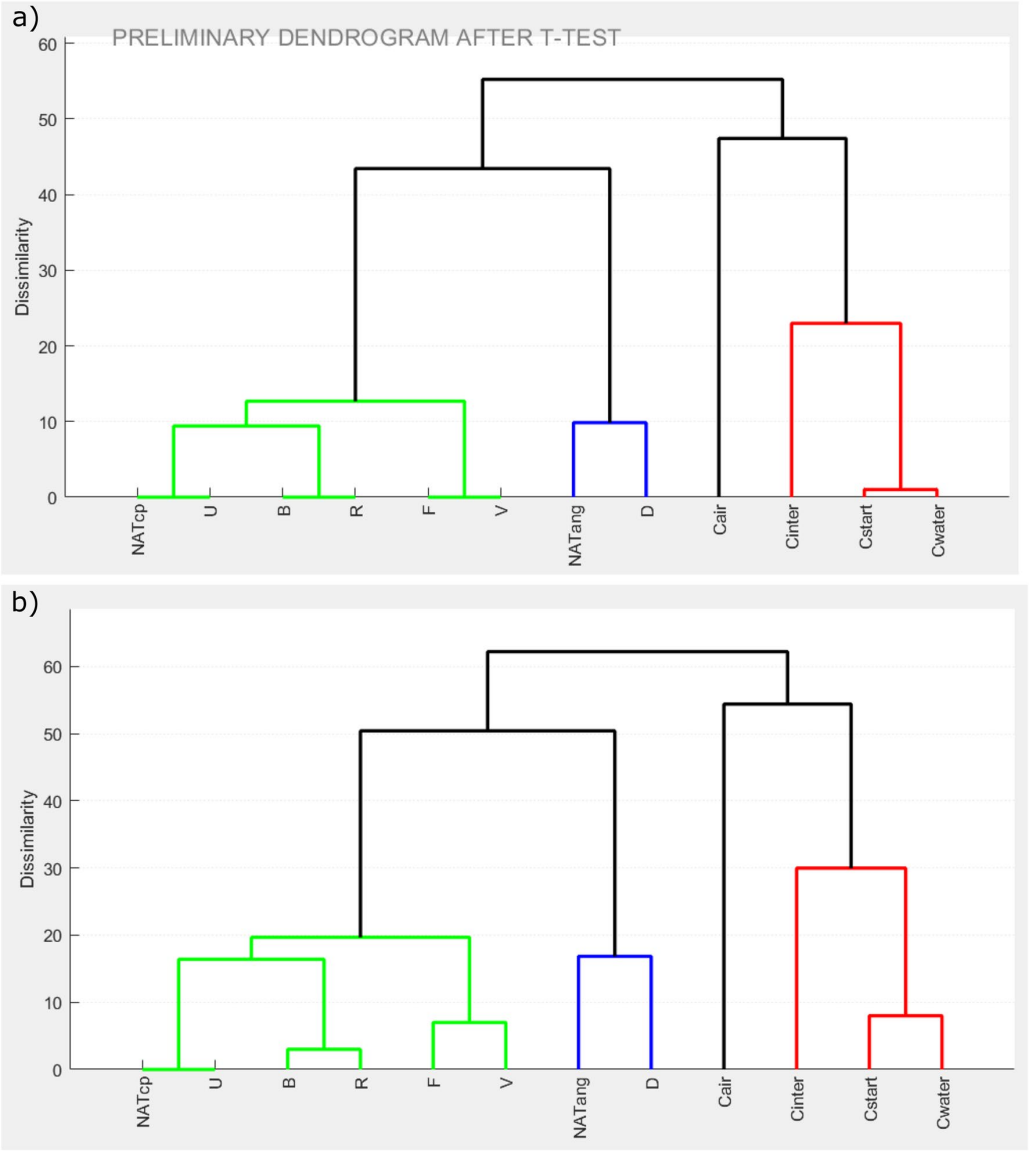
Screenshots showing the output for automatic DAPM via DendroScan. (**a**) Preliminary dendrogram considering dendrograms of all levels and t-tests. (**b**) Final display of DAPM results. In contrast to the upper diagram, results from e-tests are now also considered.

DendroScan provides the user the option to perform all steps described in the preceding section automatically. Figure 5 presents the results of such an “automatic DAPM”. Before initializing the final step (i.e., conduction of e-tests), DendroScan provides a preliminary dendrogram, to give the user a general overview of how samples are morphologically grouped after conducting T-tests (Fig. 5a). This diagram also helps the user to decide which standards to use for subsequent e-tests. For our demonstration, two different standards were available: for curvi-planar and for angular ash. According to the preliminary dendrogram all three sample pairs in question are more similar to “NATcp” than to “NATang”, which suggests that using the standards for curvi-planar ash is more suitable. Once the user has selected the standards, the DAPM is completed, and the results are displayed as a final overview dendrogram (Fig. 5b), consistent with all findings from the section above.

Information on selected shape parameters, e-test results and warnings are provided in a log file. DendroScan also automatically saves the dendrograms and final X-matrix in the “results” folder, under a newly generated sub-folder with the name “DAPM_yyyymmdd_HHMM”, based on the date and time of computation.

## Discussion

In our example, sample “U” (particles generated by IFCI with remelted dome rock and collected via U-tube) is, among all experimental samples, the only one which morphometrically fits the curvi-planar Havre ash from the eruption site (“NATcp”). Each of the other samples shows a distinctive morphometric signature, which has been revealed by DAPM. This finding is consistent with those from earlier studies^34,36^, according to which the curvi-planar Havre ash was thermohydraulically generated by IFCI processes. It further corroborates the inference that melt composition, fragmentation type and post-fragmentation cooling behaviour are important parameters in controlling the shape of the resulting fragments.

Using DendroScan to conduct a DAPM automatically is much quicker than conducting the DAPM manually by a step-by-step procedure. With DendroScan, DAPM can swiftly sort multiple data sets by morphometric dissimilarities and identify samples of statistically identical shapes. Due to the general dependency of statistical power on the number of samples analysed *N* we recommend that the number of samples tested by DAPM should be strongly limited. The DAPM technique is powerful and has been successfully tested for 22 samples^34^, but it should follow as much pre-sorting and dataset reduction as is practicable.

So far, we used all 17 available non-identical shape parameters for our DAPM demonstration. Using the default settings ensures that all morphometric nuances are considered in the analysis. This approach is recommended if the goal of the morphometric analysis is to identify samples that are statistically equivalent, or to reveal morphometric differences. However, it has to be noted that many shape parameters are not statistically independent from each other. Although mathematically defined in a different way, many of them measure a similar morphological aspect. This partial redundancy could cause a bias in *X* due to Eq. (1), and certain morphological aspects might be overrepresented in the resulting dendrogram^34^. We therefore recommend a two-fold shape parameter selection strategy: If the aim is to verify significant differences or statistical equivalences between samples, we suggest using the default setting, i.e. 17 shape parameters for DAPM. However, if the aim is to interpret the degree of morphometric dissimilarities between samples of different shape, we recommend the user to repeat the DAPM with a subset of the 17 shape parameters.

For demonstration, we study the dissimilarities between different contraction-run samples (“Cair”, “Cinter”, “Cstart”, “Cwater”, see Figs. 4 and 5), and explore how they reflect the differences in the inferred cooling curves (Fig. 1). For this purpose, the automatic DAPM was repeated using two subsets of shape parameters: (1) aspect ratio, convexity and solidity (AR_LI, Con_LI, Sol_LI, see Table 1), following the considerations of Liu et al.^31^ (2) convexity, circularity, rectangularity, form factor and Feret aspect ratio (Con_LI, Circ_SC, Rec_SC, FF_1, AR_F, see Table 1), which were found to be least redundant by Schmith et al.^30^.

**Figure 6:**
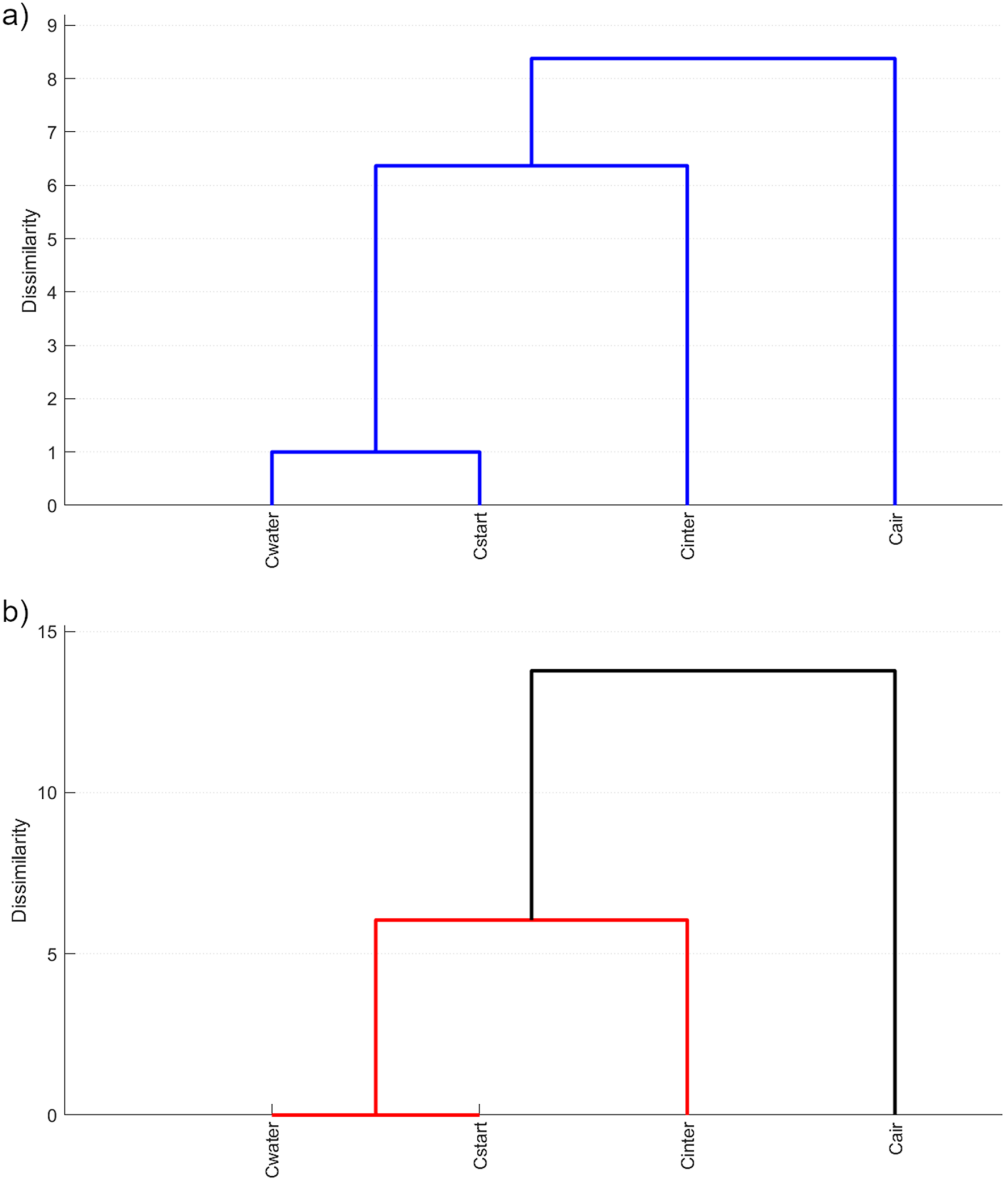
Results of DAPM with shape parameter subsets. (**a**) Dendrogram resulting from using AR_LI, Con_LI and Sol_LI. (**b**) Dendrogram produced by DAPM using Con_LI, Circ_SC, Rec_SC, FF_1 and AR_F (for notation see Table 1). Using DAPM with a limited number of shape parameters with low redundancy is recommended if the aim is to explore relative dissimilarities.

The resulting dendrograms are presented in Fig. 6. While shape parameter subset (1) resolves the differences between “Cwater” and “Cstart” (Fig. 6a), these differences are not detected when subset (2) is used (Fig. 6b). Importantly, however, although the absolute dissimilarity values differ, the over-all ranking of relative dissimilarities between samples show a consistent and systematic pattern. “Cwater” is most similar to “Cstart”, whereas “Cinter” is characterized by a slightly larger dissimilarity and “Cair” is the sample most strongly distinguished from this group. This consistency allows us to infer that the timing of cooling is more important than the amount of water used for cooling: the “Cinter” run used 600 ml of water, while only 240 ml of water was used for the generation of “Cstart”. Yet, the relatively low volume of water was enough to produce particles very similar to those completely cooled by water. As schematically illustrated in Fig. 1, the thermal gradient is significantly controlled by the timing of when melt and crucible come into contact with water. We infer that the steeper the thermal gradient, the larger the compressional forces from the contracting crucible. The subtle differences in compressional forces are reflected in the morphometric signature of the fragments and can therefore be decoded via DAPM.

DAPM has to date been applied only for shape analyses in 2D. Here it was tested exclusively against magma fragmentation processes, but we expect the presented method to be of general use in investigating processes that affect particle shape (e.g. ductile particle deformation, or abrasion). Finally, we note that while 2D analyses have been successfully applied for distinguishing among ash-particle populations and inferring eruptive styles^1–7^, measurements in 3D are increasing in response to growing accessibility of X-ray micro-tomography. 3D measurements have been shown to be more effective in some fields, such as assessing the terminal particle velocities for volcanic ash dispersion modelling^18^. Future developments of DendroScan will therefore include also the option to analyse data sets of 3D shape parameters.

## Conclusion

DendroScan offers a comprehensive set of statistical tools to comparatively analyse two or more morphometric data sets. This new open-source program provides the user with the option to sort multiple data sets according to their morphometric similarities and differences. Furthermore, it implements t-tests and e-tests, two powerful and easy-to-use statistical tools, for a pairwise comparison of data sets. These components are combined to yield a particle-morphology-test strategy, which has recently been introduced as dendrogrammatic analysis of particle morphometry (DAPM)^34^. With DendroScan we here offer the geological community a free and open source program, which in conjunction with the particle shape analysis software PARTISAN^32^ makes 2D morphometric analysis powerful, simple, fast and efficient.

## Data Availability

DendroScan and the discussed morphometric sample datasets can be downloaded at: https://github.com/lsschmidt/DendroScan (doi.org/10.5281/zenodo.4256651).
